# Lactate-mediated mixotrophic co-cultivation of *Clostridium drakei* and recombinant *Acetobacterium woodii* for autotrophic production of volatile fatty acids

**DOI:** 10.1186/s12934-024-02481-3

**Published:** 2024-07-26

**Authors:** Alexander Mook, Jan Herzog, Paul Walther, Peter Dürre, Frank R. Bengelsdorf

**Affiliations:** 1 Institute of Molecular Biology and Biotechnology of Prokaryotes, University of Ulm, Ulm, Germany; 2 Institute of Bioprocess and Biosystems Engineering, Hamburg University of Technology, Hamburg, Germany; 3 Central Facility for Electron Microscopy, Ulm University, Ulm, Germany; 4 Institute of Microbiology and Biotechnology, University of Ulm, Ulm, Germany

## Abstract

**Background:**

Acetogens, a diverse group of anaerobic autotrophic bacteria, are promising whole-cell biocatalysts that fix CO_2_ during their growth. However, because of energetic constraints, acetogens exhibit slow growth and the product spectrum is often limited to acetate. Enabling acetogens to form more valuable products such as volatile fatty acids during autotrophic growth is imperative for cementing their place in the future carbon neutral industry. Co-cultivation of strains with different capabilities has the potential to ease the limiting energetic constraints. The lactate-mediated co-culture of an *Acetobacterium woodii* mutant strain, capable of lactate production, with the *Clostridium drakei* SL1 type strain can produce butyrate and hexanoate. In this study, the preceding co-culture is characterized by comparison of monocultures and different co-culture approaches.

**Results:**

*C. drakei* grew with H_2_ + CO_2_ as main carbon and energy source and thrived when further supplemented with D-lactate. Gas phase components and lactate were consumed in a mixotrophic manner with acetate and butyrate as main products and slight accumulation of hexanoate. Formate was periodically produced and eventually consumed by *C. drakei*. A lactate-mediated co-culture of the *A. woodii* [P_*bgaL*__*ldhD*_NFP] strain, engineered for autotrophic lactate production, and *C. drakei* produced up to 4 ± 1.7 mM hexanoate and 18.5 ± 5.8 mM butyrate, quadrupling and doubling the respective titers compared to a non-lactate-mediated co-culture. Further co-cultivation experiments revealed the possible advantage of sequential co-culture over concurrent approaches, where both strains are inoculated simultaneously. Scanning electron microscopy of the strains revealed cell-to-cell contact between the co-culture partners. Finally, a combined pathway of *A. woodii* [P_*bgaL*__*ldhD*_NFP] and *C. drakei* for chain-elongation with positive ATP yield is proposed.

**Conclusion:**

Lactate was proven to be a well-suited intermediate to combine the high gas uptake capabilities of *A. woodii* with the chain-elongation potential of *C. drakei.* The cell-to-cell contact observed here remains to be further characterized in its nature but hints towards diffusive processes being involved in the co-culture. Furthermore, the metabolic pathways involved are still speculatory for *C. drakei* and do not fully explain the consumption of formate while H_2_ + CO_2_ is available. This study exemplifies the potential of combining metabolically engineered and native bacterial strains in a synthetic co-culture.

**Supplementary Information:**

The online version contains supplementary material available at 10.1186/s12934-024-02481-3.

## Background

Volatile fatty acids (VFAs) are essential commodity chemicals, serving both as standalone products and as building blocks for a variety of applications in the food, pharmaceutical and polymer industries [1]. At industrial scale, acetate and butyrate are primarily produced via chemical synthesis from crude oil or natural gas [2]. Conversely, to produce hexanoate, anaerobic fermentation is the commercially favored process [3]. The carboxylate platform process operates with undefined open consortia of microorganisms, predominantly yielding VFAs through primary and secondary fermentations from organic waste streams [4]. Although such open consortia enable a highly flexible and robust platform process, product spectra and yields are limited by the species present and the abundance of respective cells. These cell numbers can only indirectly be steered by inoculation and process conditions [5]. Another approach is the establishment of defined bacterial co-cultures with well understood strains for narrower, more specific, and therefore easier steerable processes [6]. The synthesis of butyrate, hexanoate, and the corresponding alcohols from syngas (H_2_, CO, CO_2_) by *Clostridium autoethanogenum* and *Clostridium kluyveri* is a well-documented example of such a synthetic co-culture [7]. Additionally, genetic engineering has the potential to enable novel biotransformations and platform processes by sharing the metabolic burden between two or more bacterial strains with different capabilities. The synthetic co-culture of an engineered lactate-producing *Acetobacterium woodii* strain and the type strain *Clostridium drakei* SL1 was proven to produce butyrate and hexanoate from H_2_ + CO_2_ in laboratory-scale bioreactors [8, 9]. In this approach, lactate served as intermediate between two strains, coupling the fast CO_2_ fixation capability of *A. woodii* [10] with the chain elongation ability of *C. drakei* [11, 12]. In addition to lactate, formate has been identified as a transient intermediate [9]. The Wood-Ljungdahl pathway (WLP) for CO_2_ fixation, present in both *A. woodii* and *C. drakei*, can prematurely terminate under energy-deficient conditions, e.g. ATP-deficiency through disruption of cation homeostasis [13]. This fact was found during lactate production [14, 15], resulting in the accumulation and subsequent export of formate. Recently, there has been a surge in interest regarding cell-to-cell interactions within co-cultures, particularly following the discovery of cell fusion events in *Clostridia*, exchanging RNA and proteins [16]. Further phenomena have been observed, including the formation of nanotubes (possibly enabling cross-feeding between *Acinetobacter baylyi* and *Escherichia coli* cells [17]) and the upregulation of the pili/flagella associated genes [18] of *C. autoethanogenum* co-cultivated with *C. kluyveri* compared to the monoculture. This study aims to characterize substrate consumption and growth of lactate- and CO_2_ + H_2_-fed *C. drakei* in mono- and co-cultures. In this regard, metabolite exchange and hints to cell-to-cell interactions of the lactate-producing *A. woodii* [P_*bgaL*__*ldhD*_NFP] strain [15] with *C. drakei* SL1 were examined in concurrent and sequential co-culture approaches.

## Methods

### Strains and cultivation

Table 1 lists the strains used in this work and their relevant features. *Acetobacterium woodii* strains were constructed as described in Mook et al. [15]. The *C. drakei* SL1 (DSM 12750) type strain was obtained from the German Collection of Microorganisms. *A. woodii* and *C. drakei* strains were cultivated in modified DSM 135 [19] with the addition of 20 µg mL^− 1^ uracil. Autotrophic and mixotrophic growth experiments were performed in rubber-sealed 500-mL Müller-Krempel flasks with 50 mL medium. The headspace was flushed seven times with N_2_ + CO_2_ (80:20) to ensure anaerobic conditions. After autoclaving, the headspace atmosphere was replaced with H_2_ + CO_2_ (67:33) for growth experiments. Initial headspace pressure for all co-cultures was 100–110 kPa and 130 kPa for *C. drakei* monocultures. When the headspace pressure dropped below 30 kPa, flasks were repressurized to 100 kPa. Before inoculation, MgSO_4_ was supplemented from an sterile anaerobic stock solution to a final concentration of 0.33 g L^− 1^. Clarithromycin (5 µg mL^− 1^) was only added for the pre-cultivation of recombinant *A. woodii* strains and omitted for growth experiments including *C. drakei* cells. For pre-cultivation, 10% DMSO tocks of the *A. woodii* strains and *C. drakei* were used to inoculate Hungate tubes with 5 mL modified DSM 135 supplemented with 40 mM fructose. Subsequently, biomass was transferred to Müller-Krempel flasks with H_2_ + CO_2_ headspace atmosphere to adapt cells to autotrophic growth conditions before they were used as inoculum in subsequent growth experiments. The strains were cultivated at 30 °C, moreover Müller-Krempel flasks were continuously shaken at 130 rpm. When needed, gene expression of the P_*bgaL*_ controlled gene encoding the lactate dehydrogenase-FAST fusion protein (NFP) was induced by addition of 6.8 g L^− 1^ lactose. Co-cultures with the empty plasmid control strain *A. woodii* [p83] were also supplemented with lactose to ensure comparability. None of the used strains showed consumption of lactose.

**Table 1:** Bacterial strains used in this work and their notable features

Strain	Genotype	Description	Source
*A. woodii ∆lctBCD ∆pyrE*	*Acetobacterium woodii ∆lctBCD ∆pyrE*	*A. woodii* DSM 1030 mutant with deleted lactate dehydrogenase complex, uracil auxotroph	Mook et al. 2022 [15]
*A. woodii* [p83]	*Acetobacterium woodii ∆lctBCD ∆pyrE* [pMTL83251]	*A. woodii* mutant carrying the empty backbone plasmid pMTL83251, erythromycin resistant	Mook et al. 2022 [15]
*A. woodii* [P_*bgaL*__*ldhD*_NFP]	*Acetobacterium woodii ΔlctBCD ΔpyrE* [pMTL83251_P_*bgaL*__NFP]	*A. woodii* mutant capable of lactate production and FAST-mediated fluorescence via the lactate dehydrogenase-FAST fusion protein (NFP)	Mook et al. 2022 [15]
*C. drakei*	*Clostridium drakei* SL1	DSM 12750 type-strain	DSMZ

### Analytics

During growth experiments, up to 2 mL cell suspension were withdrawn via syringes to measure OD_600_ and metabolic end products. Headspace pressure of the Müller-Krempel flasks was determined via a handheld manometer before each sampling procedure. Differences of headspace pressure between sampling points were summed up and plotted as pressure loss. The OD_600_ was determined using the GENESYS 30 vis spectrophotometer (Thermo Fisher Scientific Inc., Waltham, MA, USA). For the analysis of metabolic products, cell suspensions were centrifuged at 17,968 × *g* at 4 °C for 20 min. Part of the resulting supernatant was prepared for gas chromatography (GC) and part for high-performance liquid chromatography (HPLC).

Lactate and acetate concentrations in general were determined using the Agilent 1260 Infinity II HPLC system (Agilent Technologies, Santa Clara, CA, USA) with a diode array detector for detection. A 150 × 8 -mm column packed with a polystyrene divinylbenzene copolymer was used for separation of 20 µL supernatant per injection. The column was heated to 40 °C and 5 mM H_2_SO_4_ was used as mobile phase with a flow rate of 0.7 mL min^− 1^. Data analysis was performed with the OpenLab CDS ChemStation Edition A.01.03 software package (Agilent Technologies, Santa Clara, CA, USA). Butyrate, hexanoate, and ethanol concentrations were determined using either a Clarus 600 or a Clarus 680 gas chromatograph (PerkinElmer, Inc., Waltham, MA, USA), both equipped with a 30 m x 0.32 mm Elite FFAP capillary column (30 m x 0.32 mm) (PerkinElmer, Inc., Waltham, MA, USA). For GC analytics, 480 µL of the sample supernatant and external standards were acidified by mixing with 20 µL of 2 M HCl, respectively. 1 µL of HCl-mixed supernatant was injected via an auto-sampler with the injection temperature set to 225 °C. The temperature profile for the separation started with 2 min at 80 °C, followed by a ramp-step up to 190 °C at 10 °C min^− 1^ and a further ramp-step to 250 °C at 40 °C min^− 1^. The final temperature was held for 1 min. Detection was facilitated with a flame ionization detector at 300 °C with the detector gasses H_2_ (45 mL min^− 1^) and synthetic air (450 mL min^− 1^, N_2_:O_2_ 79.5:20.5). For the comparison of lactate-mediated and autotrophic co-cultures, acetate concentrations were determined via gas chromatography as described above.

### Fluorescence based methods

The measurement of fluorescent cells, mediated by the fluorescence-activating and absorption-shifting tag protein (FAST) [20] produced by *A. woodii* [P_*bgaL*__*ldhD*_NFP] was performed as described earlier [15]. FAST-mediated fluorescence of cell suspensions was measured using a SYNERGY H1 microplate reader (BioTek, Bad Friedrichshall, Germany) and the fluorogen ^TF^Lime at a final concentration of 10 µM. Fluorescence readouts were normalized against the OD_600_ of the harvested and washed cells.

### Scanning electron microscopy

Monocultures of *A. woodii* cells and *C. drakei* cells as well as the co-culture of both strains were cultivated to harvest cells for scanning electron microscopy. The cells were grown similarly as the other co-culture growth experiments also performed in this work. After 66 h of cultivation (Additional file 1, Fig. S1) a total of 4 mL of each respective cell suspension was harvested by centrifugation at 7,711 x g for 10 min at 4 °C. The cell pellets were suspended in 1 mL fixation solution (0.2 M phosphate-buffered saline (PBS, pH 7.3), 5% (v/v) glutaraldehyde, 2% (w/v) sucrose). Fixed cells were five times washed with 0.01 M PBS before final fixation with 2% (v/v) aqueous osmium tetroxide. Afterwards, cells were stepwise dehydrated with propanol and afterwards block-stained in 1% (w/v) uranyl acetate. Finally, cells were completely dehydrated by critical-point-drying and coated with platinum. The Hitachi S-5200 UltraHigh Resolution FE scanning electron microscope (Hitachi, Ltd. Corp., Chiyoda, Japan) was used for cell visualization.

## Results

### Monoculture *C. drakei*

*C. drakei* was grown using H_2_ + CO_2_ from the headspace atmosphere. Additionally, D-lactate was supplemented at three different starting concentrations (0 mM, 20 mM and 40 mM), to investigate its utilization under autotrophic and mixotrophic growth conditions. All *C. drakei* cells were inoculated in triplicates from H_2_ + CO_2_ and D-lactate-adapted precultures.

**Fig. 1:**
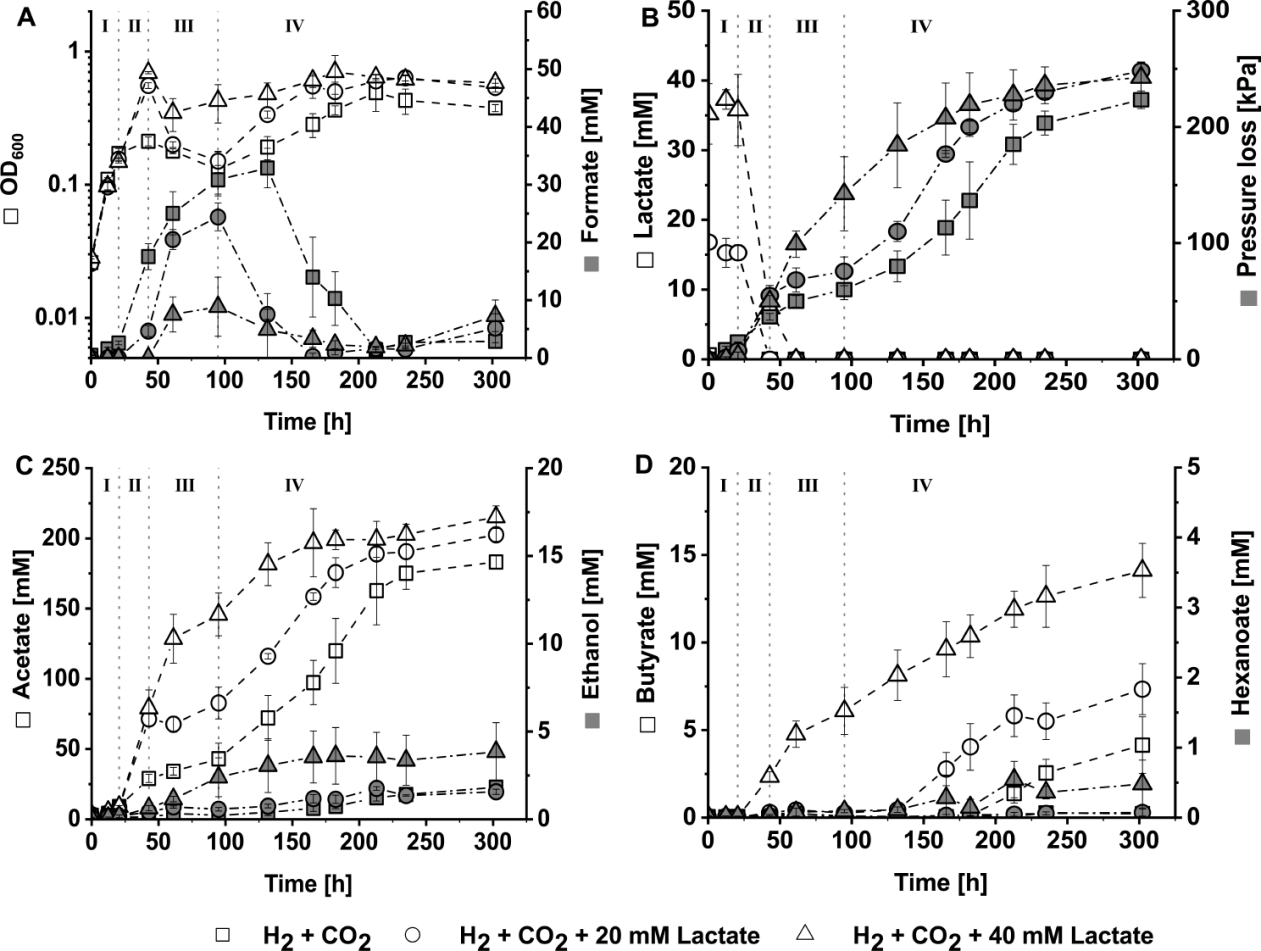
Autotrophic and mixotrophic growth experiments with *C. drakei* employing H_2_ + CO_2_ as headspace atmosphere with different lactate supplementation. (**A**), optical density (white) and formate concentration (grey); (**B**), substrate consumption with lactate concentration (white) and accumulated pressure loss of the headspace (grey); (**C**), short-chain products acetate (white) and ethanol (grey); (**D**), butyrate (white) and hexanoate (grey). (squares □): Autotrophic *C. drakei* cultures with 110 kPa H_2_ + CO_2_ in the headspace and no lactate supplementation. (circles ○): Mixotrophic *C. drakei* cultures with 110 kPa H_2_ + CO_2_ in the headspace and supplementation of 20 mM lactate. (triangles ∆): Mixotrophic *C. drakei* cultures with 110 kPa H_2_ + CO_2_ in the headspace and supplementation of 40 mM lactate. Plotted data points are the arithmetic mean of three biological replicates. Error bars represent the respective standard deviation

The growth pattern of these three *C. drakei* monoculture approaches were divided into four phases. Depending on the approach cells reached respective phases with slight temporal delay (Fig. 1). In phase I, between 0 h and 21 h of cultivation, cells in all three approaches potentially grew using yeast extract as primary substrate and without significant lactate or H_2_ + CO_2_ consumption. In the same timeframe, the mean OD_600_ of the three monoculture approaches increased from about 0.03 ± 0.001 to roughly 0.11 ± 0.01 (Fig. 1A) with an avarage specific growth rate of 0.006 h^− 1^. In phase II, between 21 h and 43 h of cultivation, lactate was quickly depleted by cells in the 20 mM and 40 mM lactate-supplemented approaches, together with consumption of gas phase components (Fig. 1B). The OD_600_ for the 20 mM and 40 mM lactate-supplemented approaches peaked at 0.56 ± 0.03 and 0.69 ± 0.01, respectively, while cells of the approach without lactate-supplementation reached an OD_600_ of 0.21 ± 0.02 in this phase. The specific growth rates in phase II were 0.024 h^− 1^ for cells of the 40 mM lactate-supplemented approach, 0.018 h^− 1^ for cells of the 20 mM lactate supplemented approach, and 0.002 for cells of the non-lactate-supplemented approach. Curiously, in phase II formate was accumulated by cells of the non-lactate-supplemented and the 20 mM lactate-supplemented approaches (Fig. 1A). In phase III, from 43 h to 95 h, H_2_ + CO_2_ from the headspace was the primary substrate, with volumetric uptake rates mirroring available biomass (Fig. 1B). During this phase, OD_600_ in all three monoculture approaches either remained stationary or decreased, while formate was further accumulated (Fig. 1A). The final phase IV, between 95 h and 302 h of cultivation, was characterized by the combined consumption of gas phase and depletion of the previously accumulated formate before cells entered a stationary phase. Cells of both the non-lactate-supplemented and the 20 mM lactate-supplemented approaches exhibited strong growth after declining in phase III, reaching optical densities of 0.49 ± 0.14 and 0.60 ± 0.07 respectively (Fig. 1A). The OD_600_ of the 40 mM lactate-supplemented culture reached 0.64 ± 0.02 in the same timeframe.

Lactate concentrations in the medium, as well as headspace pressure were monitored to account for substrate uptake of the cells (Fig. 1B). For all three monoculture approaches, lactate was mainly consumed in phase II with a maximum volumetric consumption rate of 1.28 mM h^− 1^ for the 40 mM lactate-supplemented approach. The uptake of H_2_ + CO_2_ from the headspace was also initiated in phase II (Fig. 1B). While lactate was still available, the accumulated pressure loss rate peaked at 2.1 kPa h^− 1^ for the 20 mM lactate-supplemented approach and 2.4 kPa h^− 1^ for the 40 mM supplemented one. In the absence of lactate, gas uptake began earlier, leading to a volumetric pressure loss rate of 0.8 kPa h^− 1^. After 132 h, during the second growth phase of the non-lactate-supplemented cells, a maximum volumetric pressure loss rate of 1.3 kPa h^− 1^ was attained. During the second growth phase of the 20 mM lactate-supplemented cells, a maximum volumetric pressure loss rate of 1.8 kPa h^− 1^ was reached. The 40 mM lactate-supplemented approach, where the second growth phase was not as pronounced, showed a maximum volumetric pressure loss rate of 1 kPa h^− 1^, leading to a total accumulated pressure loss of 243 ± 13 kPa.

Acetate was the main product for all investigated monocultures (Fig. 1C). *C. drakei* cells grown without lactate supplementation produced 183.1 ± 4.9 mM acetate after 302 h, with the volumetric production rate peaking at 0.9 mM h^− 1^ in phase II. Cells in the 20 mM lactate-supplemented approach on average produced 202.6 ± 4.8 mM acetate during fermentation, with the maximum volumetric production rate of 2.83 mM h^− 1^ in phase II. The 40 mM lactate-supplemented approach exhibited the highest volumetric production rate in the same timeframe with 3.1 mM h^− 1^, leading to a final acetate concentration of 215.1 ± 8.1 mM. Small amounts of ethanol were produced in all three approaches (Fig. 1C), with a maximum of 3.9 ± 1.7 mM produced by cells in the 40 mM lactate-supplemented culture.

The natural capability of *C. drakei* for chain-elongation was of special interest for further co-cultivation experiments, thus butyrate and hexanoate production were tracked throughout the fermentations. All three approaches produced butyrate with the final concentration increasing depending on the amount of supplemented lactate, resulting in 4.1 ± 1.6 mM, 7.3 ± 1.5 mM and 14.1 ± 1.5 mM in ascending order. Interestingly, the cells in the 40 mM lactate-supplemented approach already produced butyrate in phase II, with a maximum volumetric rate of 0.12 mM h^− 1^, while the 20 mM lactate-supplemented cells took up to 166 h to produce measurable concentrations with a volumetric production rate of 0.07 mM h^− 1^. Cells of the non-lactate-supplemented approach started butyrate production around the 213 h mark with a maximum volumetric rate of 0.04 mM h^− 1^, which was also the mean volumetric production rate for the lactate-supplemented approaches. Hexanoate was produced in traces by cells in the 40 mM lactate-supplemented culture, with a noticeable onset of production at the 132 h mark. Final hexanoate titer amounted to 0.48 ± 0.34 mM with measurements having a large error margin.

During the fermentation, formate emerged as an important intermediate, that was produced and subsequently consumed (Fig. 1A). The measured peak concentrations of formate increased with decreasing lactate supplementation, from 8.9 ± 5.1 mM to 24.4 ± 2.4 mM and even 32.9 ± 3.4 mM for the non-lactate-supplemented approach. Maximum volumetric production rates varied from 0.41 mM h^− 1^ to 0.87 mM h^− 1^ for the lactate supplemented approaches and 0.67 mM h^− 1^ for the approach without lactate supplementation. Around the 95 h mark, formate was consumed in the lactate-supplemented cultures with a maximum volumetric rate of 0.34 mM h^− 1^ for the 20 mM lactate-supplemented approach and 0.07 mM h^− 1^ for the 40 mM lactate-supplemented approach. After 132 h, cells of the non-lactate-supplemented approach started formate reassimilation as well, with a volumetric consumption rate of 0.46 mM h^− 1^. Interestingly, towards the end of the fermentation the lactate-supplemented cultures once more exhibited formate production.

### Comparison lactate-mediated vs. autotrophic co-culture

**Fig. 2:**
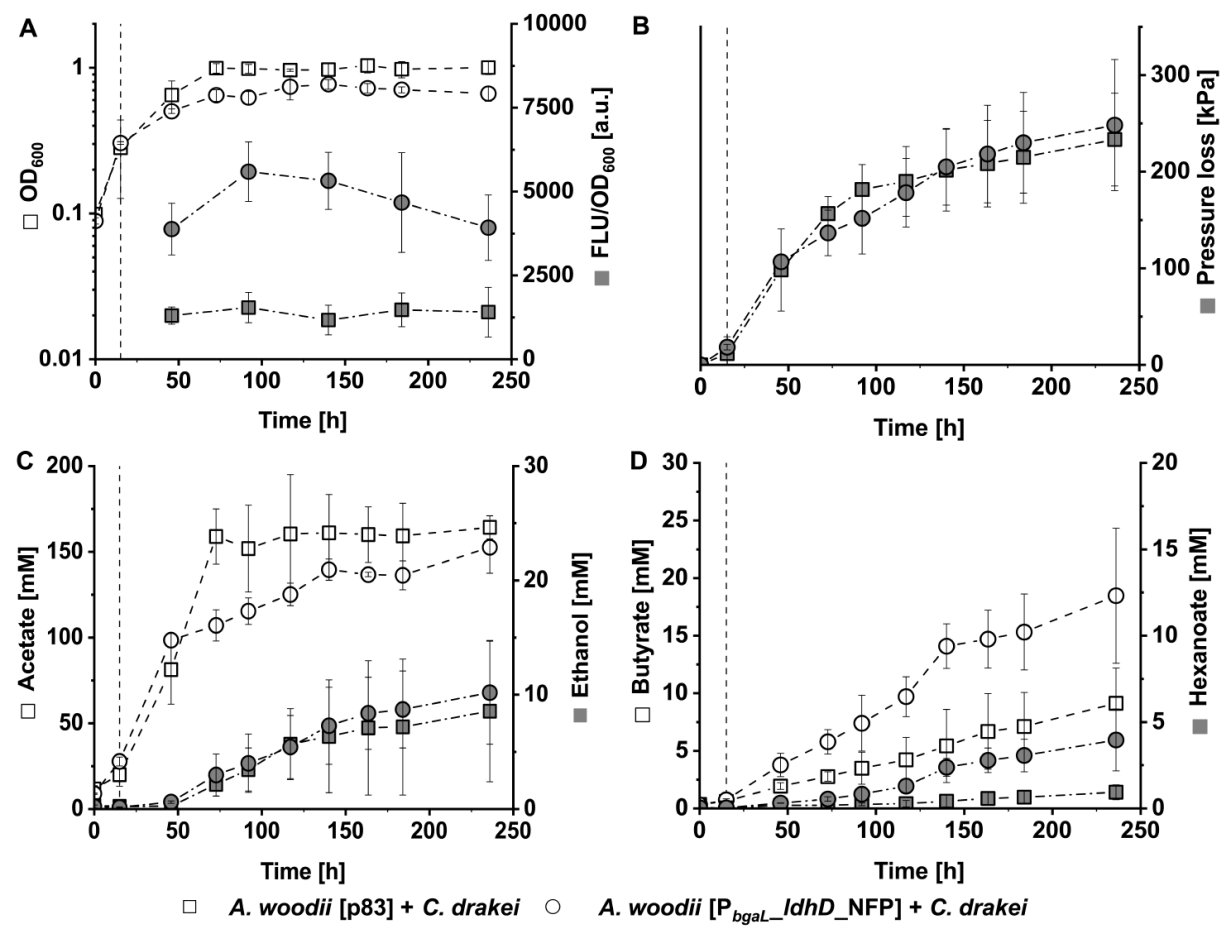
Autotrophic co-cultivation of *C. drakei* with *A. woodii* [p83] (squares □) or the lactate producer *A. woodii* [P_*bgaL*__*ldhD*_NFP] (circles ○). (**A**), optical density (white) and normalized fluorescence on whole-culture level (grey); (**B**), accumulated pressure loss of the headspace (grey); (**C**), short-chain products acetate (white) and ethanol (grey); (**D**), VFA products butyrate (white) and hexanoate (grey). Triplicate cultures were grown with 110 kPa H_2_ + CO_2_ in the headspace without antibiotics. Recombinant gene expression of *A. woodii* [P_*bgaL*__*ldhD*_NFP] was induced after 15 h, as indicated by the dashed line. Both strains were inoculated at the start of the cultivation. Plotted data points are the arithmetic mean of three biological replicates. Error bars represent the respective standard deviation

*C. drakei* cells were cultivated together with either *A. woodii* [p83] or *A. woodii* [P_*bgaL*__*ldhD*_NFP] cells in triplicate concurrent co-cultures to investigate the impact of lactate production by the latter strain in autotrophic approaches (Fig. 2). Initial cell densities for both strains were adjusted to an OD_600_ of 0.05 respectively, resulting in a starting OD_600_ of about 0.1 for both co-culture approaches (Fig. 2A). Throughout the initial cultivation phase, both co-cultures exhibited a specific growth rate of 0.012 h^− 1^ before induction of recombinant *ldhD* gene expression after 15 h, to initialize lactate production by the *A. woodii* [P_*bgaL*__*ldhD*_NFP] cells. Subsequently, the specific growth rate of these lactate-mediated co-culture cells decreased to 0.009 h^− 1^ until 46 h and further declined to 0.006 h^− 1^ until 73 h, afterwards entering a short stationary phase. The non-lactate-mediated control co-culture peaked around an OD_600_ of 0.99 ± 0.07 after 73 h and cells remained stationary until the end of fermentation at 236 h. In contrast, the cells in the lactate-mediated co-culture displayed a second minor growth phase with a specific growth rate of 0.003 h^− 1^, resulting in a peak OD_600_ of 0.77 ± 0.06 after 140 h.

While lactate concentrations in the medium could not be detected, the presence of the FAST-tagged lactate dehydrogenase (NFP) and its activity were inferred from the normalized fluorescence readings (Fig. 2A). The co-culture of *A. woodii* [p83] and *C. drakei* only showed normalized background fluorescence to an average of 1,523 ± 148 a.u. throughout the fermentation. Conversely, the cells in lactate-mediated co-culture exhibited a 2.6-fold stronger signal with 3,887 ± 783 a.u. on average and a peak reading of 5,592 ± 888 a.u. after 92 h of cultivation. Cells in both co-culture approaches consumed gas from the H_2_ + CO_2_ atmosphere, leading to an accumulated pressure loss of 233 ± 48 kPa and 248 ± 48 kPa after 236 h of cultivation. The highest volumetric pressure loss rates occurred in the stationary phase, with 2.5 kPa h^− 1^ for the control co-culture and 2.1 kPa h^− 1^ for the lactate-mediated co-culture (Fig. 2B). Conferring to the early stationary behavior, the cells in the lactate-mediated co-culture used slightly less gas between 73 h and 92 h of cultivation but caught up to the control co-culture after the second growth phase.

Acetate emerged as the primary product of fermentation in both co-culture approaches (Fig. 2C). In the control co-culture, a total of 164.3 ± 6.8 mM acetate was produced by the cells over the course of 236 h with a maximum volumetric production rate of 2.4 mM h^− 1^ between 15 h and 73 h. Subsequently, production slowed down and reached a plateau. Similarly, cells in the lactate-mediated co-culture produced 152.7 ± 15.2 mM acetate over the same 236 h period with a maximum volumetric production rate of 2.3 mM h^− 1^ between 15 h and 46 h. Interestingly, acetate production considerably slowed down the lactate-mediated co-culture compared to the control culture. No significant difference was found regarding ethanol production. Cells in both co-culture approaches produced ethanol starting around 73 h of cultivation, leading to final concentrations of 8.6 ± 6.2 mM and 10.2 ± 4.5 mM for the control and lactate-mediated co-culture approaches, respectively.

The goal of lactate-mediated co-cultivation was to enhance volatile fatty acid production. Cells of the control co-culture produced 9.1 ± 3.1 mM butyrate over the course of 236 h, with a relatively consistent volumetric production rate of 0.04 mM h^− 1^ (Fig. 2D). In contrast, cells of the lactate-mediated co-culture yielded 18.5 ± 5.9 mM butyrate during the same period. The production trajectory can be categorized in three phases. In the first phase from the start up to 117 h, the volumetric production rate was at 0.08 mM h^− 1^. Subsequently, production steeply increased to 0.19 mM h^− 1^ between 117 h and 140 h. In the final phase, a steady butyrate production with a volumetric rate of 0.05 mM h^− 1^ was realized. The lactate-mediated co-culture produced nearly 4 ± 1.8 mM hexanoate at the end of fermentation, with a maximum volumetric production rate of 0.05 mM h^− 1^ between 117 h and 140 h. Cells of the control co-culture produced hexanoate in traces, with a final concentration of 0.9 ± 0.4.

### Comparison concurrent and sequential co-culture

**Fig. 3:**
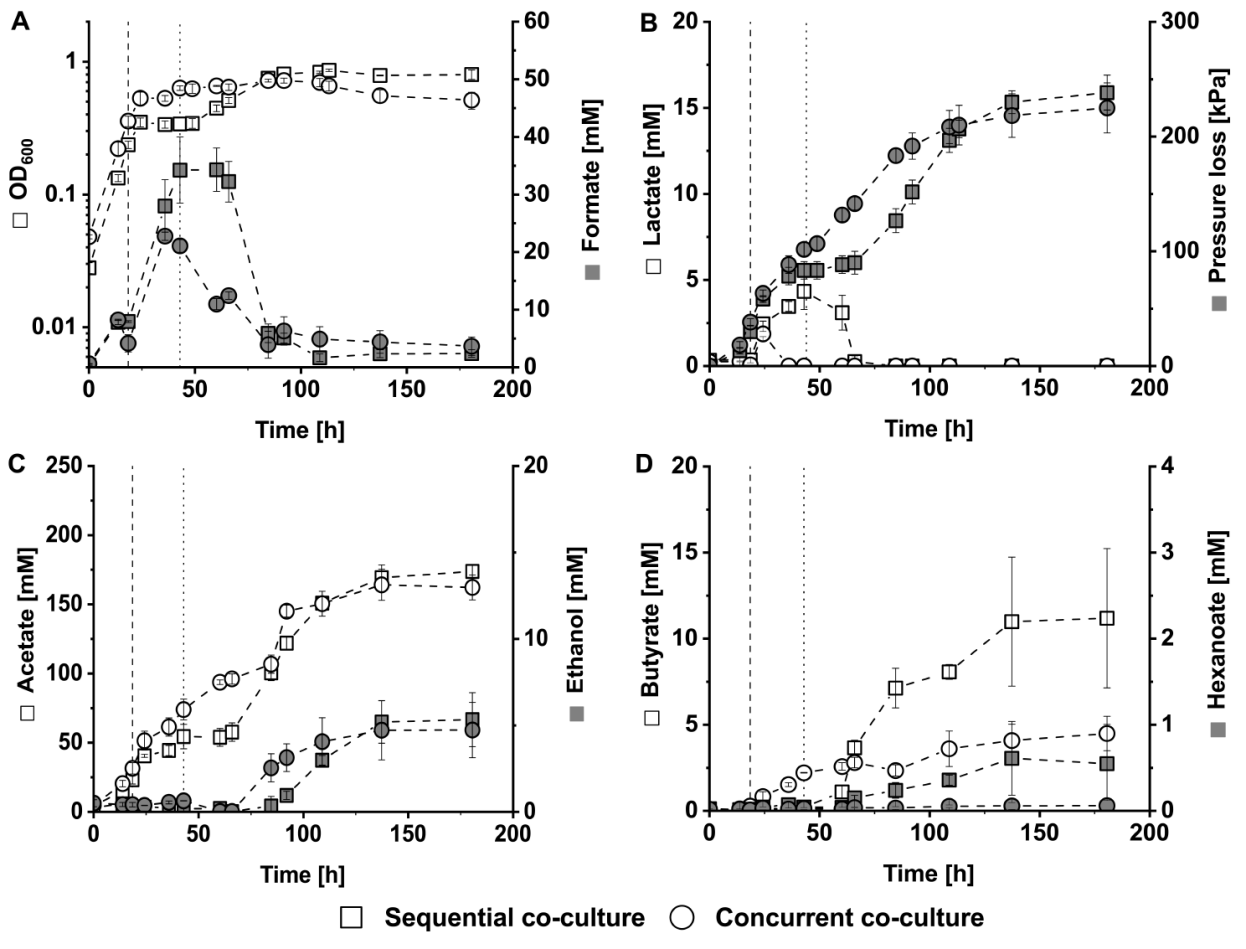
Autotrophic cultivation of *C. drakei* with *A. woodii* [P_*bgaL*__*ldhD*_NFP] either in sequential co-culture (squares □) or concurrent co-culture (circles ○). (**A**), optical density (white) and formate concentration in the medium (grey); (**B**), substrate consumption with lactate concentration (white) and accumulated pressure loss of the headspace (grey); (**C**), short-chain products acetate (white) and ethanol (grey); (**D**), VFA products butyrate (white) and hexanoate (grey). Triplicate cultures were grown with 110 kPa H_2_ + CO_2_ in the headspace without antibiotics. Recombinant gene expression of *A. woodii* [P_*bgaL*__*ldhD*_NFP] was induced after 15 h, as indicated by the dashed line. The dotted line indicates the addition of *C. drakei* from a parallel monoculture to the sequential co-culture. For the concurrent co-culture, both strains were inoculated at the start of the cultivation. Plotted data points are the arithmetic mean of three biological replicates. Error bars represent the respective standard deviation

The lactate-producing *A. woodii* [P_*bgaL*__*ldhD*_NFP] strain was cultured together with *C. drakei* in concurrent and sequential co-culture approaches to investigate possible synergistic or antagonistic interaction between the strains. The concurrent approach consisted of triplicates inoculated with *A. woodii* [P_*bgaL*__*ldhD*_NFP] and *C. drakei* cells equivalent to an OD_600_ of 0.03 respectively, resulting in a combined initial OD_600_ of roughly 0.05. The concurrent approach in this experiment is a repetition of the lactate-mediated co-culture approach seen in Fig. 2, with a lower initial OD_600_. The sequential approach was performed in two triplicates and started with monocultures of *A. woodii* [P_*bgaL*__*ldhD*_NFP] having a starting OD_600_ of 0.03 ± 0.003. These were subsequently converted to sequential co-cultures by addition of 1 mL cell suspension (corresponding to an OD_600_ of 0.004 at the most) from a *C. drakei* monoculture or the concurrent co-culture after 43 h of cultivation. As these two sequential co-cultures exhibited highly similar growth profiles, only the one started by addition of 1 mL *C. drakei* is shown in Fig. 3 while the other approach is shown in additional file 1 in Fig. S2.

Cells of the concurrent co-culture approach grew to a peak OD_600_ of 0.72 ± 0.02 after 85 h of cultivation (Fig. 3A). During the exponential growth phase, the specific growth rate was 0.029 h^− 1^. Lactate production was started by inducing the expression of the NFP-encoding gene after 19 h of cultivation, leading to a short growth stop between 24 h and 36 h. Subsequently, a second growth phase with a specific growth rate of 0.003 h^− 1^ ensued, followed by a short stationary phase and minor decline of optical density around 137 h of cultivation. The sequential co-culture showed a similar growth behavior. *A. woodii* [P_*bgaL*__*ldhD*_NFP] cells grew with a specific growth rate of 0.21 h^− 1^ reaching an OD_600_ of 0.34. Shortly after expression of the NFP-encoding gene was induced, growth paused while lactate and formate were accumulated. However, a second exponential growth phase characterized by a specific growth rate of 0.012 h^− 1^ occurred after addition of *C. drakei* cells in tandem with lactate and formate consumption. The sequential co-culture reached its peak OD_600_ of 0.86 ± 0.02 at 113 h.

*A. woodii* [P_*bgaL*__*ldhD*_NFP] cells produced lactate and consumed H_2_ + CO_2_ from the headspace (Fig. 3A). In the concurrent co-culture, lactate was measured only once, after 24 h with an average concentration of 1.9 ± 0.2 mM. In the sequential approach 4.3 ± 1.0 mM lactate was produced with a volumetric rate of 0.1 mM h^− 1^ before co-cultivation with *C. drakei* was started. Thereafter, lactate was completely consumed with a maximum volumetric consumption rate of 0.5 mM h^− 1^ within 18 h.

During the exponential growth phase, up to 24 h of cultivation, both co-culture approaches exhibited volumetric pressure loss rates of 4.3 kPa h^− 1^. Afterwards, gas consumption of the concurrent co-culture continued, resulting in a volumetric pressure loss rate of 1.9 kPa h^− 1^ up to 92 h which then leveled out until the end of fermentation. In total, the accumulated pressure loss of the concurrent co-culture approach was 225 ± 22 kPa. For the sequential co-culture approach, between 36 h and 60 h of cultivation, on average only 10 ± 8 kPa of pressure loss were observed, before the volumetric pressure loss rate increased to 2.5 kPa h^− 1^ in the second growth phase. Ultimately, the accumulated pressure loss amounted to 238 ± 15 kPa for the sequential co-culture approach.

Acetate continued to be the predominant product in both co-culture approaches (Fig. 3C), with the highest volumetric production rates occurring during the exponential growth phase. Specifically, the concurrent co-culture approach achieved a volumetric production rate of 3 mM h^− 1^, while the sequential approach reached 2.4 mM h^− 1^. Between the 60 h and 109 h marks, the concurrent co-culture maintained an average volumetric production rate of 1.3 mM h^− 1^, whereas the sequential co-culture during the second growth phase exhibited a volumetric acetate production rate of 2.1 mM h^− 1^. The final acetate concentrations were 162.2 ± 9.0 mM and 173.9 ± 4.7 mM for the concurrent and sequential approach, respectively. Both co-cultures approaches yielded around 5 mM of ethanol.

Butyrate was produced by cells in both co-culture approaches (Fig. 3D). During exponential growth, the *C. drakei* cells in the concurrent co-culture produced butyrate with a volumetric rate of 0.08 mM h^− 1^ until 43 h of cultivation. Subsequently, in the stationary phase production slowed to 0.02 mM h^− 1^ resulting in a final butyrate concentration of 4.5 ± 1.0 mM. *C. drakei* cells in the sequential co-culture produced butyrate shortly after *C. drakei* was added. The maximum volumetric production rate of 0.45 mM h^− 1^ was reached between 60 h and 66 h of cultivation, coinciding with the consumption of lactate. Afterwards, the volumetric butyrate production rate attenuated to 0.07 mM h^− 1^ resulting in an end concentration of 11.2 ± 4.0 mM. Simultaneously, the *C. drakei* cells in the sequential co-culture produced traces of hexanoate, amounting to 0.5 ± 0.5mM.

Throughout the cultivation, substantial formate production and subsequent reassimilation were observed in both approaches (Fig. 3A). In the subsequent co-culture approach, formate was produced by *A. woodii* [P_*bgaL*__*ldhD*_NFP] cells after the expression of the NFP-encoding gene was induced. In the concurrent co-culture both, *A. woodii* and *C. drakei* cells could be responsible for formate production. Thus, in the concurrent co-culture approach cells accumulated formate to a peak concentration of 22.8 ± 0.7 mM with a volumetric production rate of 0.77 mM h^− 1^. Subsequently, formate was reassimilated at a volumetric rate of 0.4 mM h^− 1^ between 43 h and 85 h, reaching a final concentration of 3.7 ± 1.6 mM. In the sequential approach, 34.2 ± 3.8 mM formate were produced by *A. woodii* [P_*bgaL*__*ldhD*_NFP] with a volumetric production rate of 0.97 mM h^− 1^. After the conversion to co-culture, formate was consumed with a maximum volumetric rate of 1.4 mM and average rate of 0.7 mM h^− 1^, resulting in a final concentration of 2.4 ± 0.6 mM.

### Microscopy

**Fig. 4:**
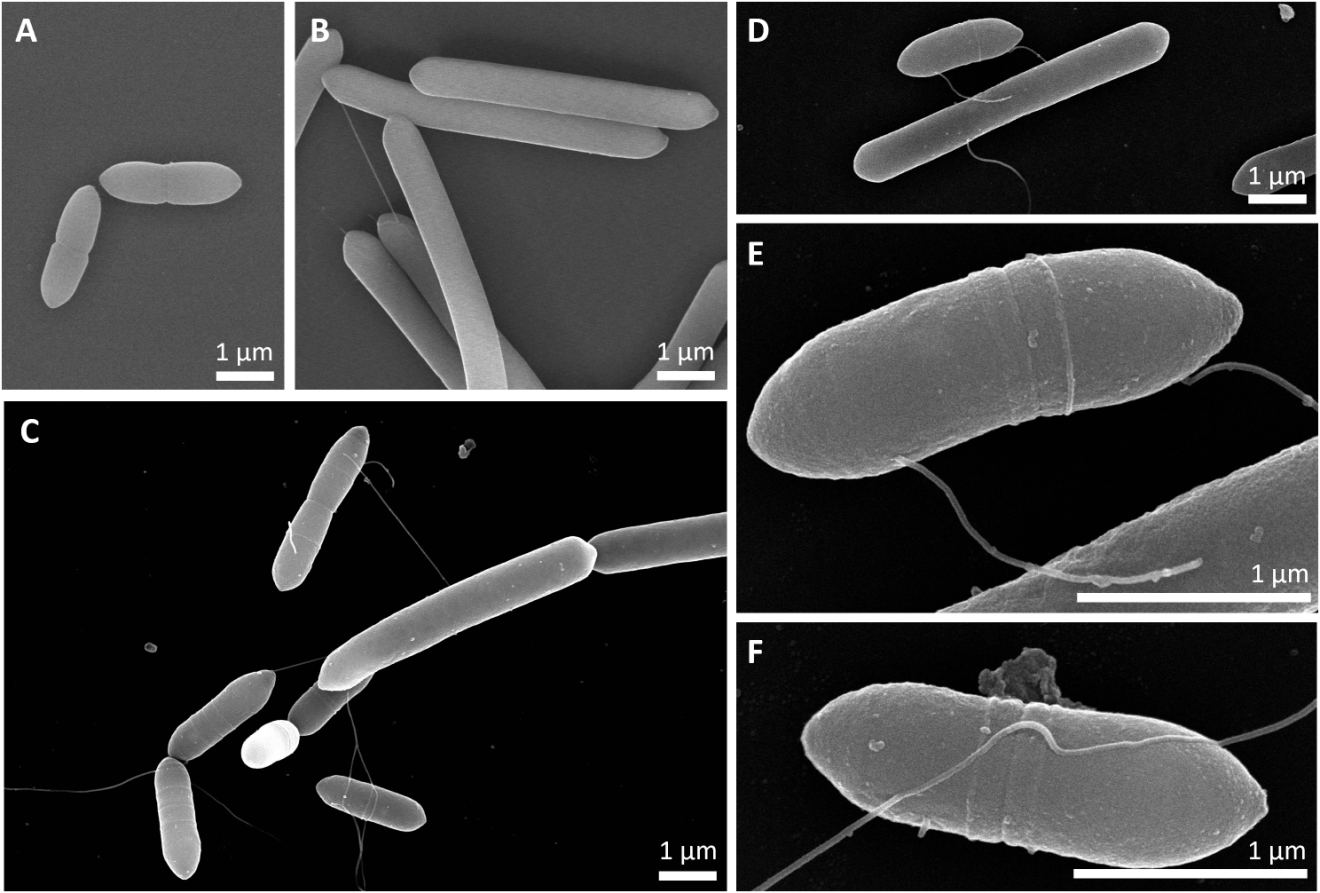
Exemplary results of scanning electron microscopy analyses. (**A**), cells from an *A. woodii* [P_*bgaL*__*ldhD*_NFP] monoculture; (**B**), cells from a *C. drakei* monoculture; (**C**-**F**), cells of *A. woodii* and *C. drakei* from a concurrent coculture, with E representing a close-up of D. 1 μm scale represented by the white bar

Scanning electron microscopy was performed to compare cell morphologies between *A. woodii* and *C. drakei* monocultures and their respective co-culture, aiming to elucidate whether interactions between cells could be monitored (Fig. 4).

**Fig. 5:**
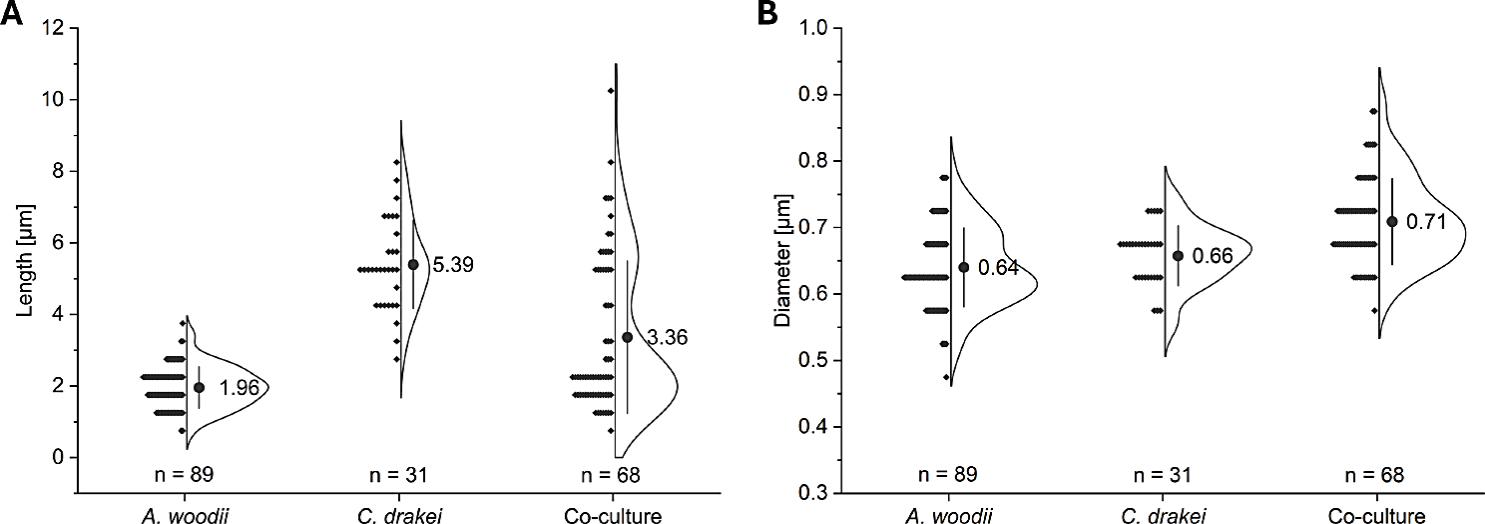
Cell measurements of mono and co-cultures of *A. woodii* [P_*bgaL*__*ldhD*_NFP] and *C. drakei* from scanning electron microscopy images. Cell length (**A**) and diameter (**B**) were only determined for fully imaged cells. The number of measured cells is given by n and their distribution symbolized by grey diamonds and the resulting idealized histogram. The respective arithmetic mean of measured lengths and diameters is symbolized by the grey dot and given next to it. Error bars represent the standard deviation. All measured cells stem from a singular biologic sample per culture

In the monoculture of *A. woodii* [P_*bgaL*__*ldhD*_NFP] (Fig. 4A), cells had an average length of 1.96 ± 0.58 µM (Fig. 5A) in the form of long oval rods. These cells had an average diameter of 0.64 ± 0.06 µM (Fig. 5B). Occasionally, singular subterminal protrusions were found on the cells, potentially representing incompletely imaged flagella. Cells were frequently divided by a constriction flanked by slightly bulged regions along the cell circumference (confer Fig. 4E). No spores were observed. Cells of the *C. drakei* monoculture (Fig. 4B) showed an average length of 5.39 ± 1.23 µM (Fig. 5). The average diameter of the long rod-shaped cells was of 0.66 ± 0.05 µM (Fig. 5B). Flagella-like filaments were oftentimes twined around the cell, and, when multiple filaments were present, they appeared arranged peritrichous. Taut filaments were also occasionally visible, forming connections between two or more cells. Same as for *A. woodii* cells, no spores were observed.

In the co-culture, both morphologies described before were also observed (Figs. 4C and 5). While the average length with 3.36 ± 2.13 µM is between those in the monocultures (Fig. 5A), the average diameter increased slightly to 0.71 ± 0.06 µM (Fig. 5B). Flagella-like filaments were more prevalent compared to the monocultures, often seemingly connecting a multitude of cells of both species (Fig. 4C-E). The exact origin of these connecting filaments, as well as the precise nature of their contact could rarely be determined. In some cases, the filament seems to be perpendicular to the cell wall (Fig. 4E), while in other cases, cells seemed to be wrapped up in it (Fig. 4C). However, in most cases the intercellular contact consisted of filaments extending along the cell surface (Fig. 4F).

## Discussion

*C. drakei* notably consumes lactate and H_2_ + CO_2_ as sole substrates [11] or mixotrophically as shown in this study. Despite preadaptation to mixotrophic growth conditions, four distinct phases of growth and substrate utilization were observed for the *C. drakei* monocultures. In phase I, the 3 g L^− 1^ of yeast extract in the medium likely served as primary substrate. Decreases of the headspace pressure and lactate concentration were minor and difficult to quantify, while the OD_600_ more than doubled in phase I. Thereafter, in phase II, D-lactate and H_2_ + CO_2_ were consumed in a mixotrophic phase until lactate was depleted. The maximum specific growth rate of 0.024 h^− 1^ in this phase was, however, lower than the 0.05 h^− 1^ reported for *C. drakei* fed solely with H_2_ + CO_2_ [12] and 0.04 h^− 1^ when grown with DL-lactate in a lab-scale bioreactor [9]. During the autotrophic phase III, after lactate depletion, H_2_ + CO_2_ were consumed while formate was further accumulated. Consumption of this formate, in combination with gas phase components, ushered phase IV, where a second growth spur was observed. As evident by the comparably slow growth of *C. drakei* when only H_2_ + CO_2_ were available in monoculture, formate and lactate seem to be favored as carbon sources.

**Fig. 6:**
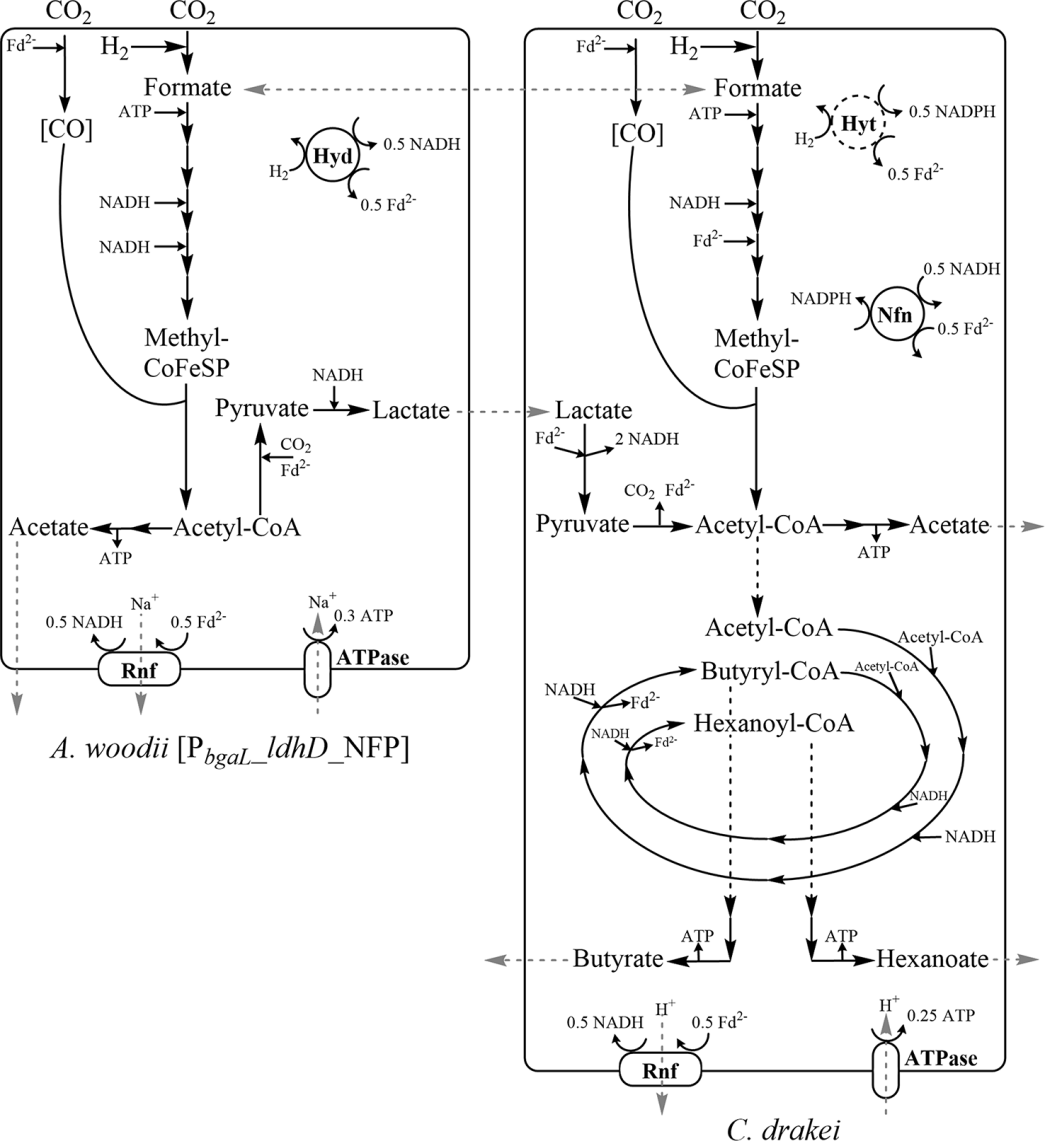
Schematic overview of central metabolism and intermediate metabolites of *A. woodii* [P_*bgaL*__*ldhD*_NFP] and *C. drakei* in co-culture. Both strains carry genes encoding for the Wood-Ljungdahl-Pathway (WLP) for carbon fixation. The simplified WLP reactions are balanced for 1 mol acetyl-CoA while the chemiosmotic energy balancing and respiratory systems are only balanced for the respective enzyme, not the total WLP. Hyt forms a complex with the formate dehydrogenase of *C. drakei* (see text). Enzyme complex abbreviations: Rnf, membrane bound Fd-NAD^+^ oxidoreductase complex; ATPase, ATP synthase; HydABC, soluble [FeFe]-hydrogenase; Hyt, NADP^+^-specific electron bifurcating [FeFe]-hydrogenase; Nfn, electron-bifurcating transhydrogenase complex; CoFeSP, corrinoid iron sulfur protein; Fd^2−^, reduced ferredoxin. Grey arrows denote extracellular transport and metabolite exchange between the two strains

A curious observation is the initial accumulation and subsequent depletion of formate in monoculture phases of *A. woodii* [P_*bgaL*__*ldhD*_NFP] and *C. drakei*, as well as, to a lesser amount, in the concurrent co-culture approach. The conversion of formate to formyl-tetrahydrofolate in the methyl branch of the WLP requires one mol ATP per mol formate. For the model acetogen *A. woodii*, the enzymes and co-factors for the WLP, respiratory and energy balancing systems are well studied (Fig. 6) [21]. In *A. woodii* the production of acetyl-CoA from H_2_ + CO_2_ via WLP and the reduction to lactate via pyruvate is ATP and NADH negative [15, 22]. *A. woodii* [P_*bgaL*__*ldhD*_NFP] does only recuperate part of the ATP invested in the methyl-branch, as part of the acetyl-CoA is pulled to pyruvate and lactate instead of the final acetogenesis step, where ATP could be generated. Thus, an ATP deficiency, caused by the induction of lactate production, leads to the observed premature stop in the WLP, resulting in accumulation and subsequent export of formate. A similar effect was shown by Schwarz et al. [13] when the artificial disruption of sodium homeostasis and the subsequent ATP deficiency led to the production of roughly 30 mM formate by resting cells of *A. woodii* DSM 1030. In the presented *C. drakei* monocultures, formate is accumulated as soon as H_2_ + CO_2_ consumption starts or lactate is completely depleted. In contrast to the engineered *A. woodii*, no ATP-negative side reaction was introduced to *C. drakei*. Thus, formate accumulation in the *C. drakei* likely does not stem from an ATP-deficiency, but some other sort of metabolic bottleneck in the WLP.

In the case of *C. drakei*, function and co-factor dependency of the central metabolism (Fig. 6) can only be assumed based on structural similarity to enzymes of other clostridial acetogens [12]. The genome of *C. drakei* contains three annotated genes encoding for possible formate dehydrogenases (Fdh). Only one of those genes clusters with other genes known to be involved in formate conversion [12]. This gene cluster encodes for an Fdh coupled to an electron-bifurcating [Fe-Fe]-hydrogenase (Hyt) such as the one initially characterized for *C. autoethanogenum*. This multifunctional Fdh-Hyt enzyme complex in vitro predominantly catalyzes the reversible reduction of CO_2_ to formate, and reduction of Fd and NADP^+^ with H_2_ as electron donor for both reactions (Fig. 6) [23]. *C. drakei* furthermore has genes for a NADH-dependent ferredoxin::NADP oxidoreductase (Nfn), originally described for *C. kluyveri* that supplies NADPH for the WLP by oxidizing NADH and Fd^2−^ [24, 25]. A final difference between the central metabolism of *C. drakei* and *A. woodii* is the methylene-THF reductase. While in *A. woodii* this enzyme is NADH dependent [21], a recently characterized variant of *Clostridium ljungdahlii*, which is the best-studied approximation for *C. drakei*, was shown to be Fd^2−^ dependent [26].

For both strains the methyl-branch of the WLP differs slightly regarding required reduction equivalents. Both species use a membrane bound Fd-NAD^+^ oxidoreductase complex (Rnf) to transport either sodium or hydrogen ions across the cytoplasmic membrane. The resulting cation moving force is used by an ATP synthase (ATPase) to generate ATP. In case of *A. woodii*, NADH and Fd^2−^ are provided via electron transfer from H_2_ in an electron-bifurcating reaction, catalyzed by a soluble [FeFe]-hydrogenase (HydABC). In case of *C. drakei*, the Fdh-Hyt complex presumably reduces CO_2_ with H_2_ to generate formate. Furthermore, the Fdh-Hyt complex can provide NADPH and Fd^2−^ by oxidation of H_2_, similar to the reaction in *C. autoethanogenum* [27]. Further balancing of reduction equivalents is mediated by the electron-bifurcating transhydrogenase complex Nfn [25]. The pyruvate reduction pathway in case of *A. woodii* [P_*bgaL*__*ldhD*_NFP] is mediated by a NADH-dependent lactate dehydrogenase while for *C. drakei* the native lactate dehydrogenase (Ldh) coupled to electron transferring flavoproteins (EtfAB) facilitates an electron-confurcating reaction when oxidizing lactate to pyruvate [28]. *C. drakei* can use acetyl-CoA, formed in the WLP or by oxidizing pyruvate, as precursor for reverse β-oxidation. The reverse β-oxidation (rBOX) pathway is encoded by the *bcs/hcs* gene cluster [29]. The rBOX pathway condenses two acetyl-CoA to one butyryl-CoA in four steps. This butyryl-CoA can be further elongated to hexanoyl-CoA by the same enzymes. The respective carboxyl-CoAs can be further converted to the respective fatty acids generating ATP.

In total, the ATP yield of the WLP together with acetogenesis for *C. drakei* under the current assumptions is 0.125 mol ATP per mol acetate (Additional file 2) compared to 0.3 mol ATP per mol acetate for *A. woodii* [21]. In the case of *C. drakei*, because of the multifunctionality of the Fdh-Hyt complex, the ATP yield is the same regardless of H_2_ + CO_2_ or formate being used as substrates. The lower amounts of formate measured in the concurrent co-culture approach and the onset of formate consumption after adding *C. drakei* to the sequential approach nevertheless hint at the metabolic significance of formate in these co-cultures. Once *C. drakei* consumes formate, a second growth phase occurs, product formation increases next to increased gas uptake. One possible avenue for *A. woodii* or *C. drakei* to obtain an energetic advantage from formate usage is oxidizing it back to CO_2_, generating either only Fd^2−^ or Fd^2−^ in combination with NADPH, respectively [23, 30]. Furthermore, coupled pathways such as the glycine-synthase-reductase pathway (GSRP) [12] or possible additional but not yet studied energy conservation mechanisms could lead to a higher ATP yield [26] in combination with formate uptake.

The lactate dehydrogenase (Ldh) of *C. drakei* associates with electron-transferring flavoproteins (EtfA/B) [12]. The oxidation of 1 mol lactate to pyruvate therefore yields 2 mol NADH through an electron-confurcating reaction with Fd^2−^ as cofactor [31]. As no ATP is needed to convert lactate to acetyl-CoA, the ATP/acetate ratio improves up to 1 when a NADH sink is available (Additional file 2). The reverse β-oxidation pathway encoded by the *bcs*/*hcs*-cluster genes represents an optimal NADH sink [29, 32, 33]. With 2 mol NADH required per mol of elongated n-acyl-CoA, NADH obviously has a dominant influence on VFA yields [34]. Lactate represents an ideal substrate as it supplies acetyl-CoA building blocks while also generating an NADH surplus. Earlier published data of *C. drakei* grown at 1-L bioreactor scale with 120 mM lactate resulted in roughly 35.6 mM butyrate and 13.4 mM hexanoate [9]. The lactate/butyrate ratio of 0.3 mol/mol in the bioreactor was comparable to the 0.39 mol/mol calculated with an initial lactate concentration of roughly 36.1 ± 1.1 mM in the 40 mM lactate supplemented *C. drakei* monoculture (Fig. 1). However, hexanoate yields of the *C. drakei* monoculture were 10-fold lower compared to the data published by Herzog et al. [9], with a lactate/hexanoate ratio of 0.1 compared to 0.01 in this study. Nevertheless, lactate mediation improved butyrate and hexanoate titers compared to the non-lactate mediated co-culture.

The internal energy and carbon metabolism is not the only deciding factor for VFA production. In a synthetic co-culture, the population dynamic between the strains needs to be considered. *A. woodii*, with its comparably high volumetric gas uptake rate [10], seems to be the dominant species at the start of the co-culture until lactate and formate production are initiated and growth slows down. Consequently, *C. drakei* appears to be the dominant partner, as implied by the secondary growth phase in the concurrent and sequential co-culture approaches. The shared metabolic burden between *A. woodii* and *C. drakei* in a lactate-mediated co-culture yielded 4 mM hexanoate and roughly 18.5 mM butyrate, comparing well to a *Clostridium* spp. dominated mixed culture in a hollow fiber bioreactor where 20.4 mM butyrate and 8 mM caproate were produced from H_2_ + CO_2_ [35] and the 6 mM butyrate and 0.9 mM hexanoate obtained in an earlier experiment with limited hydrogen supply [8].

Lower seeding densities of *C. drakei* could also explain the divergence of final titers in the different co-culture experiments. In the high-yield co-culture experiment, *C. drakei* cells equivalent to an OD_600_ of 0.05 were used for the inoculum. The concurrent co-culture cells of both strains resulted in an initial combined OD_600_ of 0.05 ± 0.002, with, at most, *C. drakei* cells equal to an OD_600_ of 0.03 used for the inoculum. For the sequential co-culture, at most, *C. drakei* cells equivalent to an OD_600_ of 0.004 were added to an *A. woodii* [P_*bgaL*__*ldhD*_NFP] monoculture with an almost 86 times higher optical density. This might also explain the slow shift from the intermediate stationary phase to the second growth phase for the sequential co-culture. Furthermore, similar OD_600_ values do not necessarily translate to the same amount of cells per mL for cells of different sizes [36]. Mixed culture approaches aiming to produce hexanoate from lactate have already demonstrated that high VFA production rates coincide with the abundance of the respective chain elongating strain [37, 38]. In contrast, the chain-elongator *C. kluyveri* in co-cultures with *C. carboxidivorans* is the lesser culture constituent [39]. While the actual abundance of *C. drakei* and *A. woodii* cells remains yet to be elucidated, an increase in *C. drakei* seeding densities could improve further co-cultivation attempts.

Synthetic co-cultures benefit from the same survival strategies as natural mixed cultures like chemotaxis [40] or the coaggregation of multiple species to form biofilms [41]. In the last few years, the search for interspecies cell-cell interactions has led to astounding observations, such as the cell wall fusion of *C. ljungdahlii* and *Clostridium acetobutylicum* cells leading to exchanges of RNA and proteins resulting in hybrid cells [16]. Upregulation of flagella/pilli genes, among others, was observed in synthetic co-cultures of *C. autoethanogenum* and *C. kluyveri* [6]. In the SEM images presented here, filaments were observed to form connections between the long *C. drakei* cells and the short *A. woodii* cells. The genome sequences of both strains [12, 42] include gene clusters for flagella and type IV pili. Therefore, the genetic potential to establish cell-cell contact is given. However, *A. woodii* in monoculture did not exhibit filaments as pronounced as in the co-culture. While pili and flagella, in contrast to nanotubes, are not suited to transfer intracellular components, the fixation of *A. woodii* and *C. drakei* in close proximity to each other should favor diffusive processes [43], such as extracellular electron transfer mediated by the exchange of formate [44]. Ishii et al. observed flagellum-like filaments such as the ones visible in Fig. 4 as a precursor to coaggregation of the thermophile *Pelotomaculum thermopropionicum* and the hydrogenotrophic methanogen *Methanothermobacter thermoautotrophicus.* They furthermore observed the formation of exopolysaccharides in the intracellular space of cell aggregates [43]. This observation was not made for the co-culture of *A. woodii* and *C. drakei*, with shaking of culture flasks possibly discouraging this second step of coaggregation.

## Conclusion

In this study, *C. drakei* was shown to, in monoculture, consume lactate and H_2_ + CO_2_ in tandem, with a strong preference for lactate over gaseous components. In the early stages of cultivation, formate was produced as a secondary product and eventually depleted when only H_2_ + CO_2_ were available. Depending on the supplied lactate concentrations, increasing amounts of butyrate and hexanoate could be produced by the *C. drakei* monocultures. The influence of lactate supply on VFA yields was also shown in autotrophic co-cultures with lactate generated by an *A. woodii* mutant, with up to 4 ± 1.7 mM of hexanoate produced. A second co-cultivation experiment implied that sequential co-cultivation, as opposed to concurrent co-cultivation, could possibly further improve VFA yields. Theoretical pathways for an ATP-positive VFA production in co-culture were also considered. Finally, electron microscopy of mono- and co-culture cells revealed interspecies cell-to-cell contact, implying a possible advantage of proximity between the cells.

### Electronic supplementary material

Below is the link to the electronic supplementary material.

Additional file 1: Supporting figures

Additional file 2: ATP yield calculations for *C. drakei*

## Data Availability

The datasets used and/or analyzed in this study are available from the corresponding author on reasonable request.
