# Supplementary material for: Lactate-mediated mixotrophic co-cultivation of Clostridium drakei and recombinant Acetobacterium woodii for autotrophic production of volatile fatty acids
Source: Microb Cell Fact. 2024 Jul 26;23:213. doi: 10.1186/s12934-024-02481-3 (PMC11282840; doi:10.1186/s12934-024-02481-3)
Supplement: Supplementary file 1 — Additional file 1: Supporting figures [file 12934_2024_2481_MOESM1_ESM.pdf]

Additional file 1

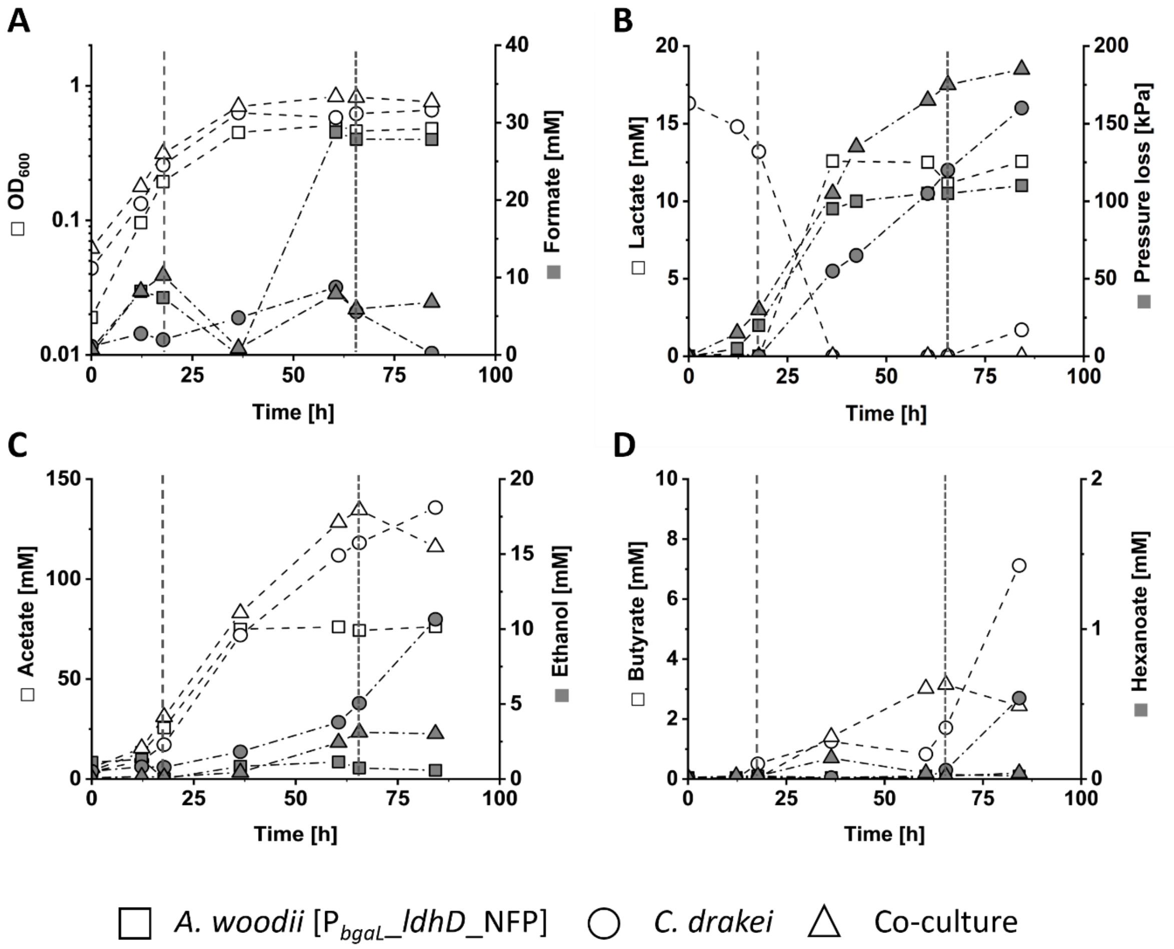

Fig. S1: Autotrophic growth experiments to generate biomass for electron microscopy. (A), optical density (white) and formate concentration (grey); (B), substrate consumption with lactate concentration (white) and accumulated pressure loss (grey); (C), product concentrations of acetate (white) and ethanol (grey); (D), product concentrations of butyrate (white) and hexanoate (grey). (Squares □), autotrophic *A. woodii* [P_{bgaL}_*ldhD*_NFP] culture. (Circles ○), mixotrophic *C. drakei* culture with H_2 + CO_2 in the headspace and lactate supplementation. (Triangles Δ), co-culture of *C. drakei* and *A. woodii* [P_{bgaL}_*ldhD*_NFP]. The dashed line indicates addition of 20 mM lactose to induce recombinant *ldhD_NFP* gene expression. The dotted line represents the time of cell harvest.

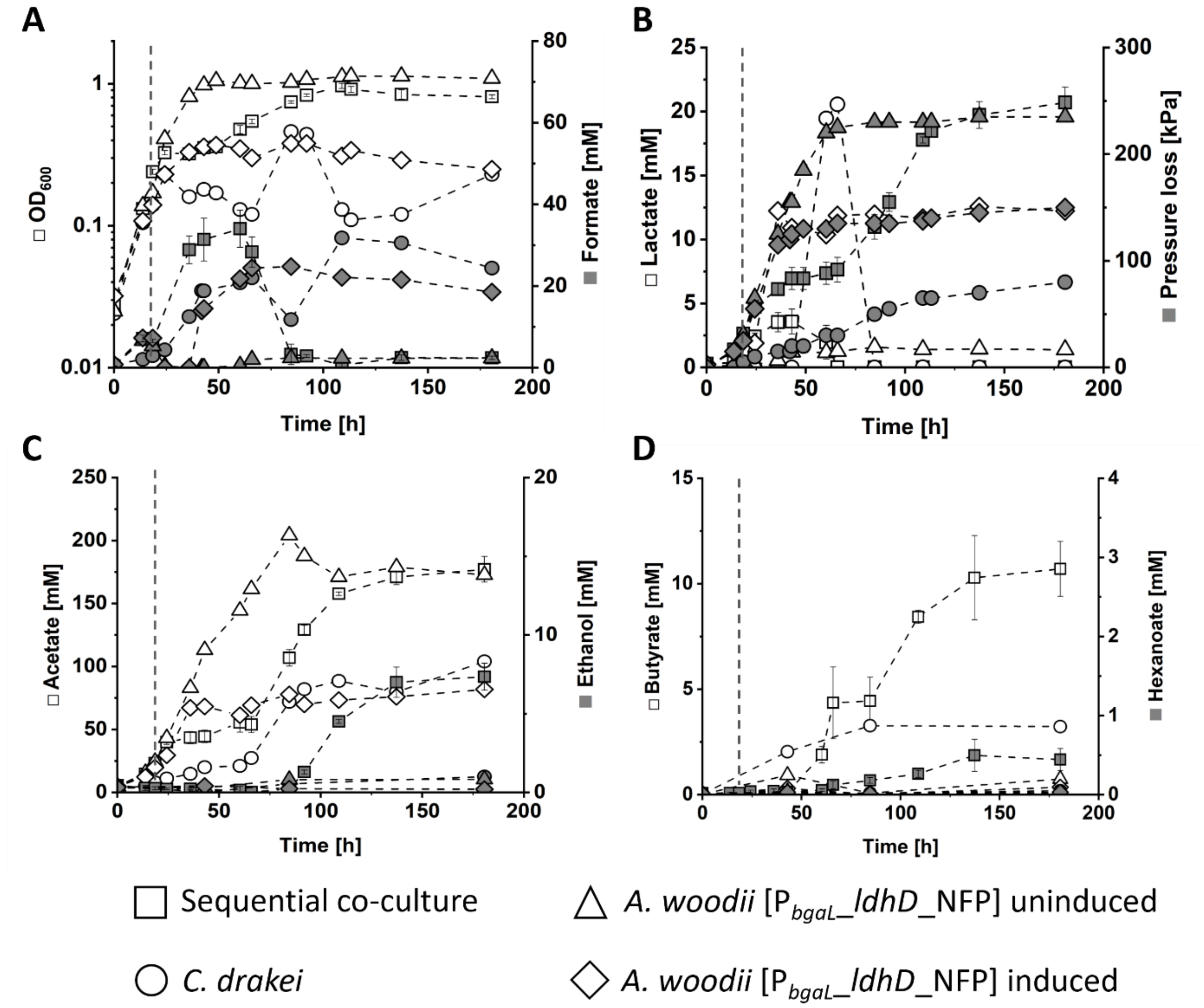

Fig. S2: Autotrophic and mixotrophic cultures of *A. woodii* [P_{bgaL}_*ldhD*_NFP], *C. drakei* and co-cultures thereof*,* employing H_2 + CO_2 as headspace atmosphere. (A), optical density (white) and formate concentration (grey); (B), substrate consumption with lactate concentration (white) and accumulated pressure loss (grey); (C), product concentrations of acetate (white) and ethanol (grey); (D), butyrate (white) and hexanoate (grey). (Squares □), triplicate of sequential co-culture transitioned by addition of 1 mL of concurrent co-culture broth. (Circles ○), mixotrophic *C. drakei* culture with 110 kPa H_2 + CO_2 in the headspace and supplementation of 20 mM lactate after 60 h of cultivation. (Triangles Δ), *A. woodii* [P_{bgaL}_*ldhD*_NFP] monoculture without induction of recombinant *ldhD_NFP* gene expression. (Diamonds ◊), *A. woodii* [P_{bgaL}_*ldhD*_NFP] monoculture without induction of recombinant *ldhD_NFP* gene expression. The dashed line indicates addition of 20 mM lactose to induce recombinant *ldhD_NFP* gene expression. For the sequential co-culture, plotted data points are the arithmetic mean of three biological replicates. Error bars represent the respective standard deviation.
